# Antimicrobial resistance, virulence genes and biofilm formation in *Enterococcus* species isolated from milk of sheep and goat with subclinical mastitis

**DOI:** 10.1371/journal.pone.0259584

**Published:** 2021-11-15

**Authors:** Mona A. El-Zamkan, Hams M. A. Mohamed

**Affiliations:** 1 Faculty of Veterinary Medicine, Department of Food Hygiene and Control, South Valley University, Qena, Egypt; 2 Faculty of Veterinary Medicine, Department of Microbiology, South Valley University, Qena, Egypt; Universidad Autonoma de Chihuahua, MEXICO

## Abstract

This study is designed to discuss the antimicrobial resistance, virulence determinants and biofilm formation capacity of *Enterococcus* spp. isolated from milk of sheep and goat with subclinical mastitis in Qena, Egypt. The obtained isolates were identified by the VITEK2 system and 16S rDNA sequencing as *E*. *faecalis*, *E*. *faecium*, *E*. *casseliflavus* and *E*. *hirae*. Overall, *E*. *faecalis* and *E*. *faecium* were the dominant species recovered from mastitic milk samples. The antimicrobial susceptibility test evidenced multidrug resistance of the isolates against the following antimicrobials: oxacillin (89.2.%), followed by vancomycin (75.7%) and linezolid (70.3%). Also, most of these isolates (73%) could form biofilms. For example, 18.9% of *Enterococcus* strains formed strong biofilm, whereas 32.4% of isolates formed moderate biofilm and 21.6% of isolates formed weak biofilm. The most prevalent resistance genes found in our isolates were *blaZ* (54%), *vanA* (40%), *ermB* (51.4%), *tetM* (13.5%) and *optrA* (10.8%). Moreover, *asa1* (37.8%), *cylA* (42.3%), *gelE* (78.4%), *esp* (32.4%), *EF3314*(48.6%) and *ace* (75.5%) were the most common virulence genes. A significant correlation was found between biofilm formation, multidrug resistance and virulence genes of the isolates. This study highlights several aspects of virulence and harmfulness of *Enterococcus* strains isolated from subclinical mastitic milk, which necessitates continuous inspection and monitoring of dairy animals.

## Introduction

Mastitis is a major concern that impacts the global dairy industry. It affects dairy ruminants’ mammary glands’ health, which is vital for milk yield, quality, and animal health. Also, it frequently necessitates significant management activities leading to massive financial losses. Consequently, it jeopardizes the huge dairy economic entities and rural areas where milk, cheese, and other dairy products are essential for the local economies [1,2]. Mastitis is classified as clinical or subclinical inflammation based on the presence or absence of symptoms. Economically, Subclinical Mastitis (SCM) is usually considered more critical to the dairy industry not only because of the hidden symptoms but additionally because milk production does not increase after full recovery from the condition [3], causing persistent economic loss. Subclinical mastitis likely occurs 15 to 40 times more frequently than clinical mastitis and is longer-lasting and it was found to persists even after treatment using antibiotics that keep it from acquiring the clinical form [4]. Therefore, SCM serves as a reservoir of pathogens that spread the udder infection among animals and is considered a public health risk.

A diversity of microbial organisms primarily causes mastitis. *Enterococcus* genus is one of the environmental causative agents of mastitis [5,6]. *Enterococcus* genus is a part of the intestinal microbiota of humans, animals and birds [7] and is prevalent in high loads in foods, especially those of animal origin [8,9]. *Enterococcus* genus is one of the opportunistic pathogens and responsible for most nosocomial infections, which are hospital-acquired ones like urinary endocarditis, tract infections, intra-abdominal and pelvic infections, surgical infections, catheter infections and central nervous system infections [10].

The massive use of antimicrobials in treating mastitis has contributed to developing the antimicrobial resistance crisis [11]. *Enterococcus* genus is intrinsically resistant to several antimicrobial agents such as aminoglycosides and β-lactams antibiotics [12]. Also, it acquired resistance against tetracyclines, ciprofloxacin, erythromycin, linezolid, daptomycin, quinupristin/ dalfopristin, and vancomycin [13]. Their existence may act as an intensive grouping of resistant genes that can be disseminated to other commensal or even pathogenic bacteria [14]. Thus, it has attained great importance in clinical microbiology.

Another major factor contributing to the severity of *Enterococcus* infections is their virulence. There are over ten different virulence genes have been identified in clinical isolates, including aggregation substance, cytolysin activator, gelatinase, enterococcal surface protein, collagen-binding protein, hyaluronidase and two newly discovered surface proteins encoded by *asa1*, *cylA*, *gelE*, *esp*, *ace*, *hyl* and *EF3314* were identified [15,16]. Similar virulence genes associated with human infections could be identified in isolates belonging to the food of animal origin [17–22].

*Enterococcus* spp. can accumulate multiple genetic elements encoding virulence traits and antibiotic resistance genes and develop biofilm that has a prominent role in *Enterococcus* infections [23]. The biofilm of *Enterococcus* promotes tolerance to harsh environmental conditions and contributes significantly to persistence during infection and food processing environment, causing ecological contamination [24,25]. Additionally, biofilms produced by *Enterococcus* spp. increase their inherent and acquired resistance to antibiotics [23], posing a significant challenge to infection treatment, especially in virulent strains.

Although different *Enterococcus* spp. were isolated from clinical and subclinical mastitis of sheep and goats [5,26–30], information regarding its biofilm production, virulence traits and the effect of different virulence genes on its ability to produce biofilm is scarcely available. As a result of the great significance of SCM and the public health importance of *Enterococcus* spp. as a leading cause of nosocomial infections and the role of their virulence genes and biofilm in these infections and their chronicity, this study aimed to investigate the role of *Enterococcus* spp. as a causative agent of subclinical mastitis in sheep and goats, its ability to produce biofilm and the role of this milk in the transmission of multidrug-resistant (MDR) and virulent *Enterococcus* spp.

## Materials and methods

### Isolation and identification of *Enterococcus* spp.

#### Ethical approval

The study was approved by the Animal Ethics Committee for Veterinary Research, Faculty of Veterinary Medicine, South Valley University, Qena, Egypt. Written informed consents were obtained from the owner(s) of the animals used in the study.

#### Sample collection and examination

A total of 115 clinically healthy sheep and goats (65 sheep and 50 goats) owned by smallholders in Qena, Egypt, were inspected for SCM by California Mastitis Test (CMT). The samples were collected depending on the availability of the sheep and goats and the permission from their owners. Sampling was performed after washing the udder with clean water and soap and teats antisepsis using 70% ethyl alcohol. Then, CMT was implemented on a separate milk sample per each mammary gland (Two samples per animal). Among these animals, 47 animals (21 sheep and 26 goats) were CMT positive. Positively reacted milk samples (30 and 38 from sheep and goat, respectively) were collected under aseptic conditions and transferred under refrigeration to the lab to be bacteriologically investigated for *Enterococcus* species. Ten μL of milk were inoculated in BHI broth and incubated for 24 hours at 37°C before being streaked on Bile Aesculin Azide Agar plates (Oxoid, CM0888) and incubated for 24 hours at 37°C. *Enterococcus* spp. were identified using VITEK 2 System (Version 08.01, bioMérieux, USA).

#### 16S rDNA sequencing and phylogenetic analysis

To validate the VITEK 2 findings, we subjected four *Enterococcus* isolates (three were clearly defined and one yielded low discrimination) to 16S rDNA sequencing after PCR amplification with the 16S rDNA primer (specified in S1 Table).

*DNA extraction and PCR amplification*. DNA extraction QIAamp DNA Mini kit (Qiagen GmbH, Hilden, Germany) was used to extract DNA from isolates with modifications from the manufacturer’s recommendations. In brief, 200 μL of the isolate suspension was incubated with ten μL of proteinase K and 200 μL of lysis buffer at 56°C for 10 min. Then, 200 μL of 100% ethanol was added to the mixture. Each mixture was then washed and centrifuged following the manufacturer’s recommendations. Nucleic acid was eluted with 100 μL of elution buffer provided in the kit.

PCR amplification targeting 16S rDNA primer was performed in Applied Biosystem 2720 thermal cycler. PCR mixture final volume (25 μL) consisted of 12.5 μL of Emerald Amp Max PCR Master Mix (Takara, Noji-higashi Kusatsu, Japan), one μL of each primer, six μL of DNA template, and then the volume was completed by PCR water. The PCR products were separated by electrophoresis on 1.5% agarose gel (Applichem GmbH, Darmstadt, Germany).

*16S rDNA sequencing and phylogenetic analysis*. PCR products were purified using the QIAquick PCR Purification kit (Qiagen, Valencia, CA). For the sequence reaction, the Bigdye Terminator V3.1 cycle sequencing kit (Perkin-Elmer) was used, and then it was purified using the Centrisep spin column. DNA sequences were obtained by Applied Biosystems 3130 genetic analyzer (HITACHI, Tokyo, Japan). All the obtained 16S rDNA gene sequences were submitted to the BLAST® analysis tool (Basic Local Alignment Search Tool) [31] on the GenBank (http://blast.ncbi.nlm.nih.gov/Blast.cgi?PROGRAM = blastn&PAGE TYPE = Blast Search & LINK LOC = blasthome#) to establish sequence identity to GenBank accessions. The phylogenetic tree was constructed by the MegAlign module of Lasergene DNAStar version 12.1 [32], and phylogenetic analysis was implemented using maximum likelihood, neighbour-joining and maximum parsimony in MEGA 7 [33].

### Phenotypic antimicrobial resistance

The antibiotic sensitivity profile of all isolated *Enterococcus* spp. was determined using penicillin (PEN, 10 units) (CT0043B), oxacillin (OX, 1 μg) (CT0159B), erythromycin (ERY, 15 μg) (CT0020B), tetracycline (TET, 30 μg) (CT0054B), vancomycin (VAN, 30 μg) (CT0058B), linezolid (LZD, 30 μg) (CT0020B), nitrofurantoin (NIT, 300 μg) (CT0036B). Oxoid, Thermo Fisher Scientific, Basingstoke, United Kingdom, supplied all commercially available antimicrobial sensitivity discs. Briefly, bacterial suspensions at a concentration of 10^5^ CFU/mL were inoculated on Mueller-Hinton agar plates and discs were placed on the surface of the medium, then incubated 24 to 48 hours at 37°C. Zones of inhibition and susceptibilities were measured and calculated according to Clinical Laboratory Standards Institute (CLSI) [34].

### PCR detection of antimicrobial resistance and virulence genes

β-lactams antibiotics resistance gene (*bla*Z) along with other drug-resistant genes to the following antibiotics: vancomycin (*vanA*), tetracycline (*tetM*), erythromycin (*ermB*) and linezolid (*optrA*) were determined by PCR using the primers displayed in S1 Table. Furthermore, isolates were screened for the presence of *asa1*, *cylA*, *gelE*, *ace*, *EF3314*, *hyl*, and *esp* virulence genes (S1 and S2 Tables).

PCR amplification was performed in a 25- μL mixture reaction that contained the EmeraldAmp Max PCR Master Mix (Takara, Japan) (12.5 μL), 1 μL of each primer of 20 pmol concentration, water (4.5 μL) and DNA template (6 μL). An Applied biosystem 2720 thermal cycler was used to perform the reaction, and electrophoresis on agarose gel (1.5%) (Applichem, Germany, GmbH) separated the PCR amplicons.

### Biofilm formation

Biofilm assays were implemented using a previously described process [35,36] in triplicates. *Enterococcus* isolates were incubated in 10 mL of Tryptic Soy Broth (TSB) with 1% glucose for 24 hours at 37°C. Then, 20 μL of each bacterial suspension were transferred to three wells of sterile 96-well polystyrene microtiter plates holding 180 μL of TSB with 1% glucose and 200 μL of uninoculated TSB with 1% glucose broth assigned as a negative control. The microtiter plate was incubated at 37°C for 24 hours. Next, the broth was cautiously withdrawn, and the wells were washed three times with sterile phosphate-buffered saline. Biofilms were then fixed with methanol for 20 min., flicked, and air-dried in a flipped position in a warm room for about 30 min. Biofilms were stained with crystal violet (2%) for 15 min. The wells were washed twice with distilled water then dried. The dyed adherent cells were resolubilized in 150 μL of acetic acid (33%) for 30 min. without shaking at room temp. Finally, a microtiter plate reader was used to estimate the OD of each well at 570 nm. The cut-off value (ODc) = average negative control OD + (3 SD of negative control). Each *Enterococcus* isolate was characterized as one of the following phenotypes: OD < ODc denoted as non-biofilm producers, ODc < OD < 2ODc denoted as weak biofilm producers 2ODc < OD < 4ODc denoted as moderate biofilm producers and OD > 4ODc denoted as strong biofilm producers as previously described Stepanovic et al. [37].

### Statistical analysis

Statistical analysis using the Chi-square test was performed by GraphPad Prism 8 and *P<* 0.05 was regarded as statistically significant. A hierarchical cluster analysis (HCA) (joining, tree clustering) was performed to group the strains based on their similarity (determined by Pearson correlation coefficient) and cluster aggregation (based on unweighted pair-group method). The input matrix for HCA comprised phenotypically (multidrug resistance) and genotypic (virulence genes) strain characteristics. XLSTAT programme version 2021.5.1 was used to process statistical data and create graphics (Addinsoft, New York, USA).

## Results

According to CMT positive results, SCM was encountered by 32.3% and 52.1% of sheep and goats, respectively. Of the 30 and 38 subclinical mastitic milk of sheep and goat, 22 and 15 isolates were identified as *Enterococcus* spp., respectively. Cultural, morphological, and VITEK 2 findings revealed that most of the species found in the obtained isolates from both sheep and goat milk were *E*. *faecalis* (30% and 28.9%, respectively), followed by *E*. *faecium* (26.7% and 10.5%, respectively) (Table 1).

**Table 1:** Prevalence of *Enterococcus* spp. identified using the VITEK 2 system in milk samples.

Sample	No. of milk samples	*E*. *faecalis*		*E*. *faecium*		*E*. *casseliflavus*		*E*. *hirae*	
		No.	%	No.	%	No.	%	No.	%
Sheep	30	9	30	8	26.7	4	13.3	1	3.3
Goat	38	11	28.9	4	10.5	0	0	0	0
Total	68	20	29.4	12	17.6	4	5.9	1	1.5

**Fig 1:**
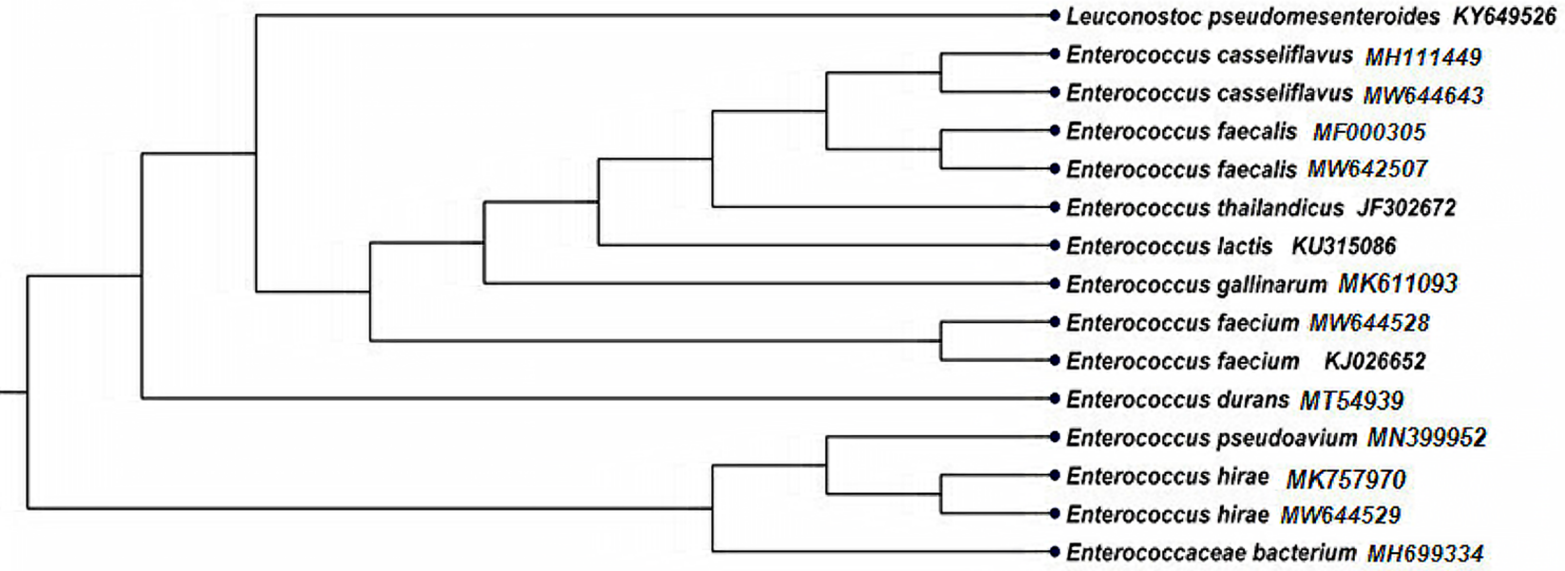
The neighbor-joining tree shows the 16S rDNA gene phylogenetic relationships of the *Enterococcus* strains isolated from subclinical mastitic milk of sheep and goat and phylogenetically related reference strains on GenBank.

The VITEK 2 system correctly identified 36 isolates with a probability ranged from 95–99% but failed to identify the correct species of one isolate. Representative four colonies were chosen (one isolate from each well-identified species and one that failed to be identified by the VITEK 2 system), submitted to 16S rDNA sequence analysis, and blasted in the GenBank. BLAST search over the GenBank revealed that *E*. *faecalis*, *E*. *faecium*, and *E*. *casseliflavus* identified by the VITEK 2 system showed a high similarity with *E*. *faecalis* (MF000305) with identity percentage (100%), *E*. *faecium* (KJ026652) with identity percentage (99.7%) and *E*. *casseliflavus* (MH111449) with identity percentage (99.4%), confirming the VITEK 2 system identification. In comparison, the isolate that gave low discrimination showed close matching to *E*. *hirae* sequence (MK757970) (99.9%) on the GenBank. The phylogenetic tree constructed from partial 16S rDNA sequencing of four isolates obtained from sheep and goat milk evidenced that the obtained isolates showed a high similarity between each other, as shown in Fig 1. The clades of the tree showed groups belonging to the same genus. The accession numbers under which the submitted 16S rDNA sequences of the obtained isolates are accessible are listed in S3 Table.

Results displayed in Table 2 present the antimicrobial resistance regarding species of the 37 isolated strains. It was found that the highest resistance of *Enterococcus* spp. was against oxacillin (89.2.%), then vancomycin with an incidence of 75.7%, followed by linezolid (70.3%), penicillin and erythromycin (64.9% each), nitrofurantoin (43.2%) and tetracycline (18.9%). All strains included in this study had acquired resistance to at least one of the antimicrobials used. A total of 25 *Enterococcus* out of 37 strains (67.6%) displayed MDR phenotype (resistance to more than three antimicrobials from three different families).

**Table 2:** Antimicrobial resistance profiles of the four *Enterococcus* species isolated from subclinical mastitis milk.

Antimicrobials			Intermediate (IR) and resistant (R) isolates							
	*E*. *faecalis* (N = 20)		*E*. *faecium* (N = 12)		*E*. *casseliflavus* (N = 4)		*E*. *hirae* (N = 1)		Total (N = 37)	
	IR No. (%)	R No. (%)	IR No. (%)	R No. (%)	IR No. (%)	R No. (%)	IR No. (%)	R No. (%)	IR No. (%)	R No. (%)
Penicillin	0	14 (70)	0	7 (58.3)	0	2 (50)	0	1	0	24 (64.9)
Oxacillin	0	16 (80)	0	12 (100)	0	4 (100)	0	1	0	33 (89.2)
Vancomycin^a^	0	16 (80)	0	8 (66.7)	0	3 (75)	0	1	0	28 (75.7)
Erythromycin	5 (25)	13 (60)	0	8 (66.7)	1 (25)	3 (75)	0	0	6 (16.7)	24 (64.9)
Tetracycline	4 (20)	7 (35)	0	0	0	0	0	0	4(11.1)	7 (18.9)
Linezolid^a^	0	15 (75)	0	8 (66.7)	0	2 (50)	0	1	0	26(70.3)
Nitrofurantoin	0	7 (35)	0	7(58.3)	0	1 (25)	0	1	0	16 (43.2)

^a^All VR *E*. *faecalis* and *E*. *faecium* isolates, except two isolates, showed resistance to linezolid.

In this study, different six patterns of antimicrobial resistance were detected. Each pattern incorporated different antimicrobial types, and this grouping was accomplished based on the resistance exhibited by our isolates, as seen in Table 3. Three strains showed resistance to the seven antimicrobials used in this study. In total, *E*. *faecalis* displayed resistance to the six patterns of antimicrobials, whereas *E*. *faecium* and *E*. *casseliflavus* strains showed resistance to three and two patterns, respectively. Also, pattern 2 of the MDR patterns was the most prevalent one (18.9%) amongst *Enterococcus* spp.

**Table 3:** Multidrug Resistance (MDR) Phenotypes in isolated *Enterococcus* strains (N = 37).

Multidrug resistance pattern		*E*. *faecalis* (N = 20)	*E*. *faecium* (N = 12)	*E*. *casseliflavus* (N = 4)	*E*. *hirae* (N = 1)	Total (N = 37)
		No (%)	No (%)	No (%)	No (%)	No. (%)
Pattern 1	PEN, OX, VAN, ERY, TET, LZD, NIT	3 (15)	0	0	0	3^a^ (8.1)
Pattern 2	PEN, OX, VAN, ERY, LZD, NIT	2 (10)	4 (33.3)	1 (25)	-	7 (18.9)
Pattern 3	PEN, OX, VAN, LZD, NIT	2(10)	3 (25)	0	1(100)	6 (16.2)
Pattern 4	PEN, VAN, ERY, TET, LZD	3(15)	0	0	-	3(8.1)
Pattern 5	PEN, OX, VAN, ERY, LZD	3 (15)	0	1(25)	-	4(10.8)
Pattern 6	PEN, VAN, ERY, LZD	1 (5)	1 (8.3)	0	-	2 (5.4)

^a^ Two strains were strong producing biofilm (*P<* 0.05).

The molecular detection of resistance genes in the isolated strains showed that the resistance to β-lactams antibiotics (penicillin and oxacillin), vancomycin, erythromycin, tetracycline, and linezolid was linked to the presence of *blaZ* (54%), *vanA* (40%), *ermB* (51.4%), *tetM* (13.5%) and *optrA* (10.8%) genes in the identified strains, respectively (Table 4).

**Table 4:** Prevalence of antimicrobial resistance encoding genes in *Enterococcus* isolates.

Resistance genes	*E*. *faecalis* (N = 20)	*E*. *faecium* (N = 12)	*E*. *casseliflavus* (N = 4)	*E*. *hirae* (N = 1)	Total (N = 37)
	No (%)	No (%)	No (%)	No (%)	No. (%)
*bla*Z	11(55)	8(66.7)	1 (25)	0	20 (54.1)
*van*A	9 (45)	4 (33.3)	1(25)	1(100)	15(40.5)
*erm*B	12 (60)	5 (41.7)	2 (50)	0	19(51.4)
*tet*M	5 (25)	0	0	0	5(13.5)
*optr*A	3 (15)	1 (8.3)	0	0	4(10.8)

**Fig 2:**
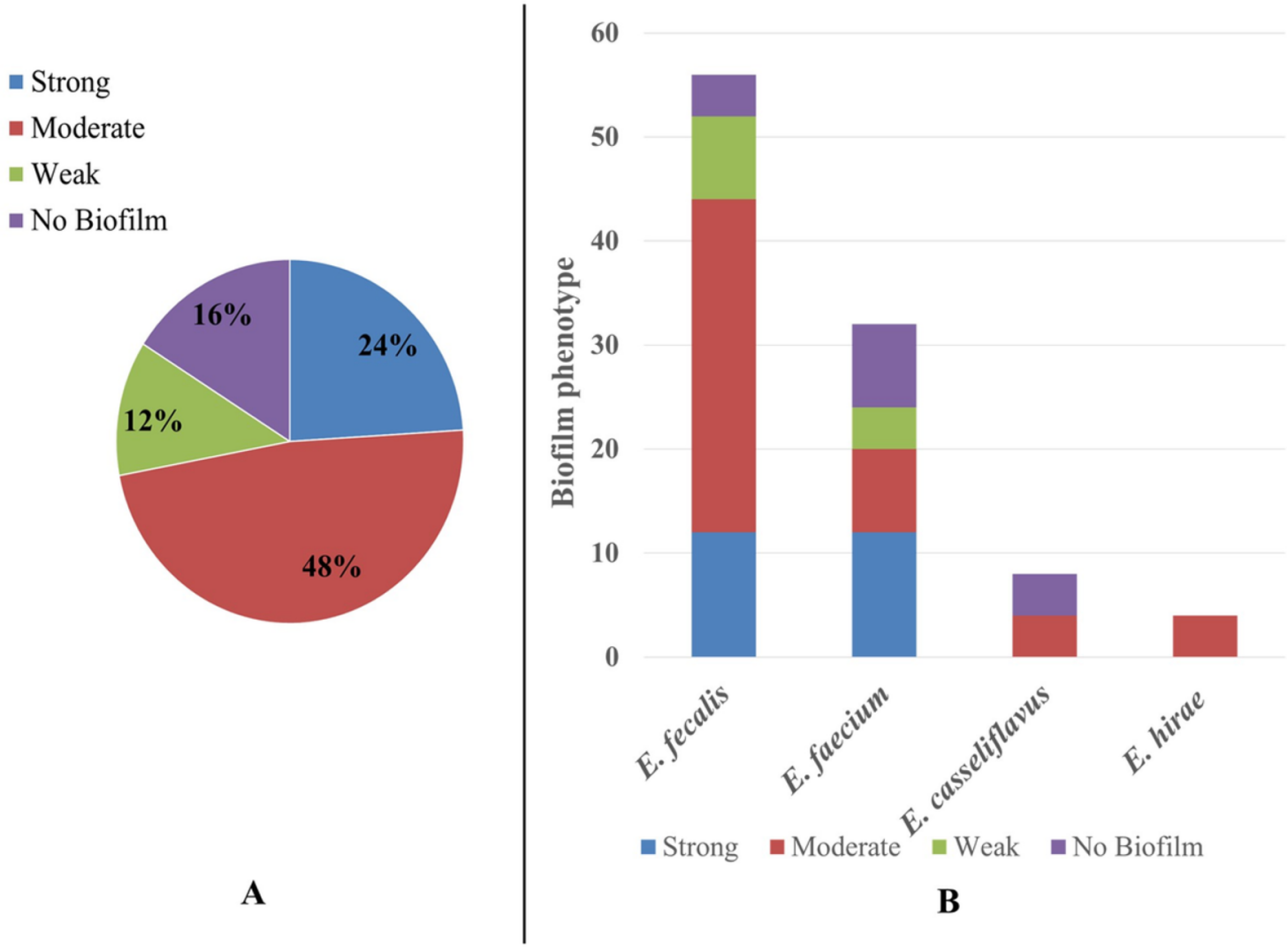
Biofilm formation by MDR *Enterococcus* isolates. A: Biofilm phenotype distribution among MDR *Enterococcus* isolates; B: Biofilm phenotype distribution among MDR isolates with reference to species.

Data illustrated in Table 5 revealed that most isolates (73%) could form biofilms at different levels. After incubation for 24 hours, seven (18.9%) *Enterococcus* strains formed strong biofilm, whereas 12 (32.4%) and eight (21.6%) *Enterococcus* isolates formed moderate and weak biofilm, respectively. Adversely, 27% of the isolates did not form a biofilm. Biofilm was produced by 84% of MDR strains, including strong (24%), moderate (48%) and weak (12%) (Fig 2A). Also, *E*. *faecalis* isolates of this study were contributed to 44% of moderate and strong biofilm of MDR strains versus 20% by *E*. *faecium* (Fig 2B).

**Table 5:** Biofilm formation by *Enterococcus* isolates^a^.

Biofilm production	*E*. *faecalis* ^b^^,^ ^c^ (N = 20)	*E*. *faecium* (N = 12)	*E*. *casseliflavus* (N = 4)	*E*. *hirae* (N = 1)	Total (N = 37)
	No. (%)	No. (%)	No. (%)	No. (%)	No. (%)
No biofilm	3 (15)	5 (41.7)	2 (50)	-	10 (27)
Weak	5 (25)	2 (16.7)	1(25)	-	8 (21.6)
Moderate	8 (40)	2 (16.7)	1(25)	1 (100)	12(32.4)
Strong	4 (20) ^**c**^	3 (25)	0	-	7 (18.9)

^a^ Twenty one out of 27 biofilm-producing *Enterococcus* isolates (77.8%) were MDR (*P<* 0.05).

^*b*^ *E*. *faecalis* that have a strong potency to form biofilm are strongly resistant to antibiotics (*P<* 0.05).

^c^ A significant relationship between subclinical mastitis and biofilm formation by isolated *Enterococcus* spp., especially *E*. *faecalis* (*P<* 0.01).

**Fig 3:**
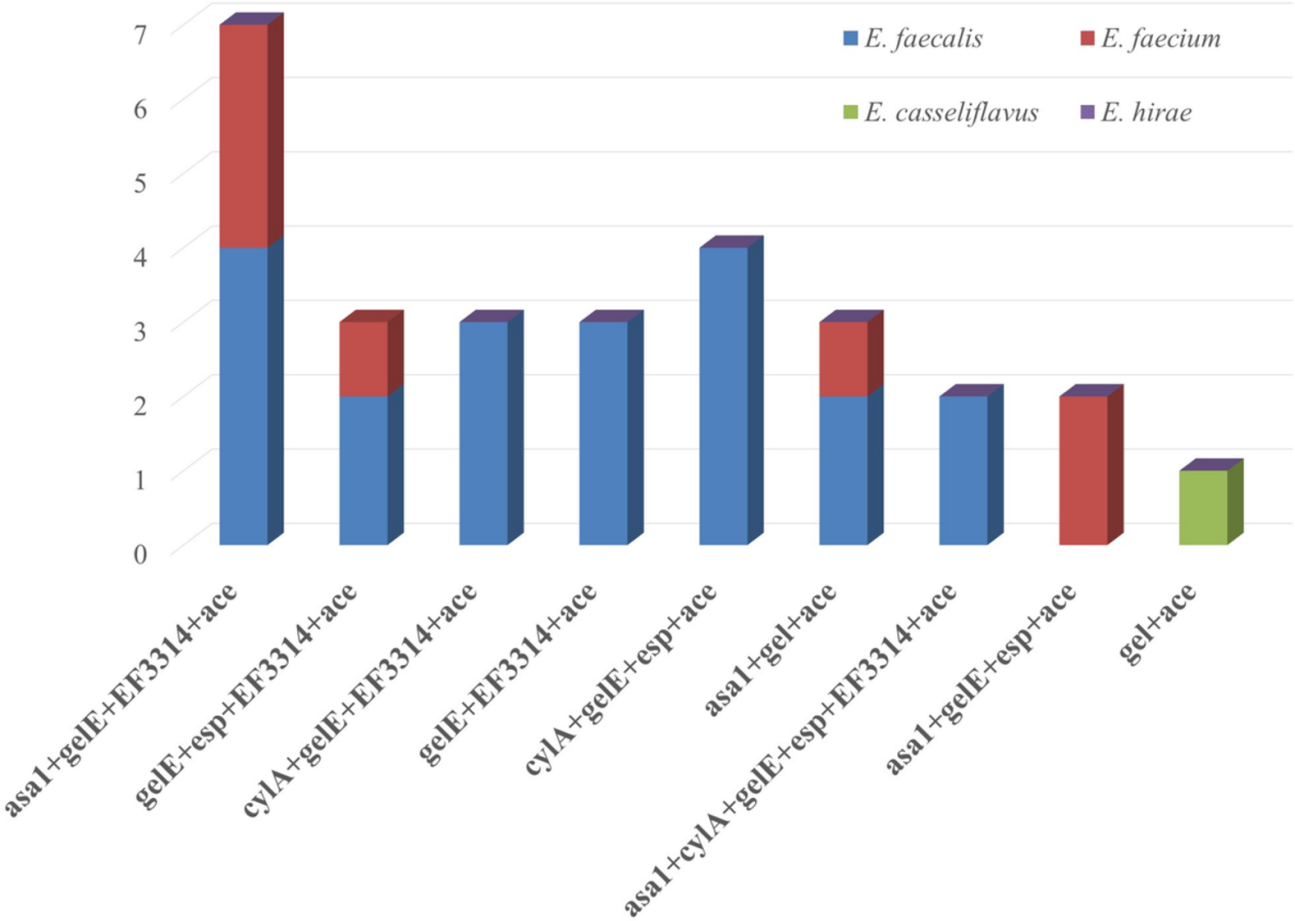
Distribution of virulence genes among different *Enterococcus* sp.

**Fig 4:**
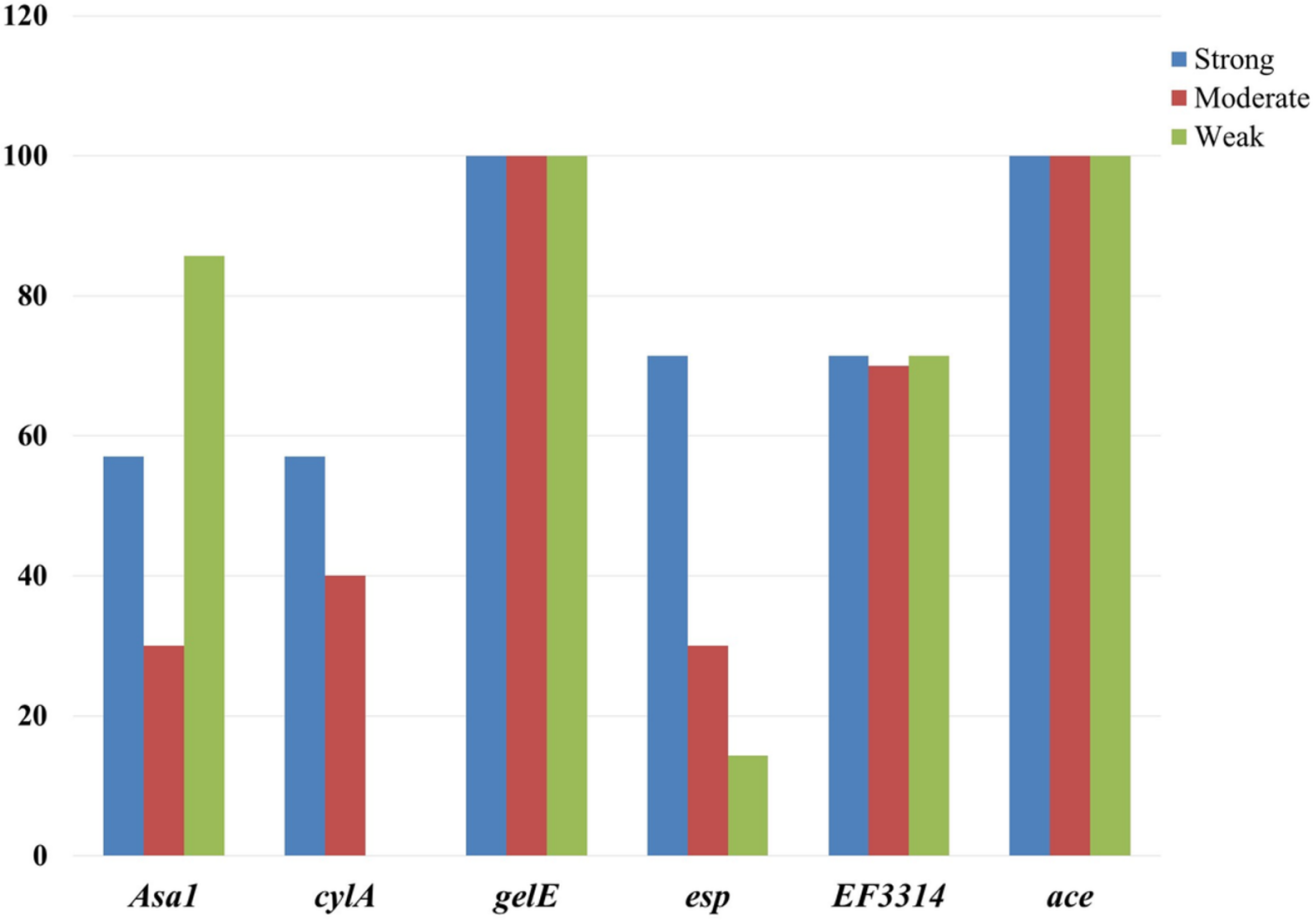
Distribution of various virulence genes among different biofilm phenotypes formed by *E*. *faecalis* and *E*. *faecium*.

The inspection for the virulence genes evidenced the presence of *asa1* (37.8%), *cylA* (42.3%), *gelE* (78.4%), *esp* (32.4%), *EF3314* (48.6%) and *ace* (75.7%) in the isolates, as shown in Table 6. However, all isolates lacked the *hyl* gene. The distribution of the virulence genes in the isolated strains revealed nine virulence profiles. Most of these profiles occurred in *E*. *faecalis* strains (Fig 3). Regarding biofilm production by virulent *Enterococcus* strains, *gelE* and *ace* genes were found in all biofilms-producing strains with its three phenotypes. At the same time, *EF3314* was found in almost 70% of the three biofilm phenotypes produced by the isolated strains (Fig 4).

**Table 6:** Prevalence and distribution of virulence genes in *Enterococcus* isolates.

Virulence genes	*E*. *faecalis* (N = 20)	*E*. *faecium* (N = 12)	*E*. *casseliflavus* (N = 4)	*E*. *hirae* (N = 1)	Total (N = 37)
	No. (%)	No. (%)	No. (%)	No. (%)	No. (%)
*asa1*	8 (40)	6 (50)	0 (0)	0	14 (37.8)
*cylA*	9 (45)	0 (0)	0 (0)	0	9 (42.3)
*gelE* ****	20 (100)	7 (58.3)	1 (25)	1 (100)	29 (78.4)
*esp* *	8 (40)	3 (25)	1 (25)	0	12 (32.4)
*EF3314* **	14(70)	4(33.3)	0(0)	0	18 (48.6)
*hyl*	0 (0)	0(0)	0 (0)	0	0 (0)
*ace* ****	20 (100)	7(58.3)	1(25)	0	28 (75.7)

***A highly significant correlation between biofilm formation and *gelE*, *ace* genes (*P*< 0.0001).

**A significant correlation between biofilm formation and the *EF3314* gene (*P*< 0.01).

* Lack of strict association between the presence of *esp* and biofilm formation (*P*> 0.05).

**Fig 5:**
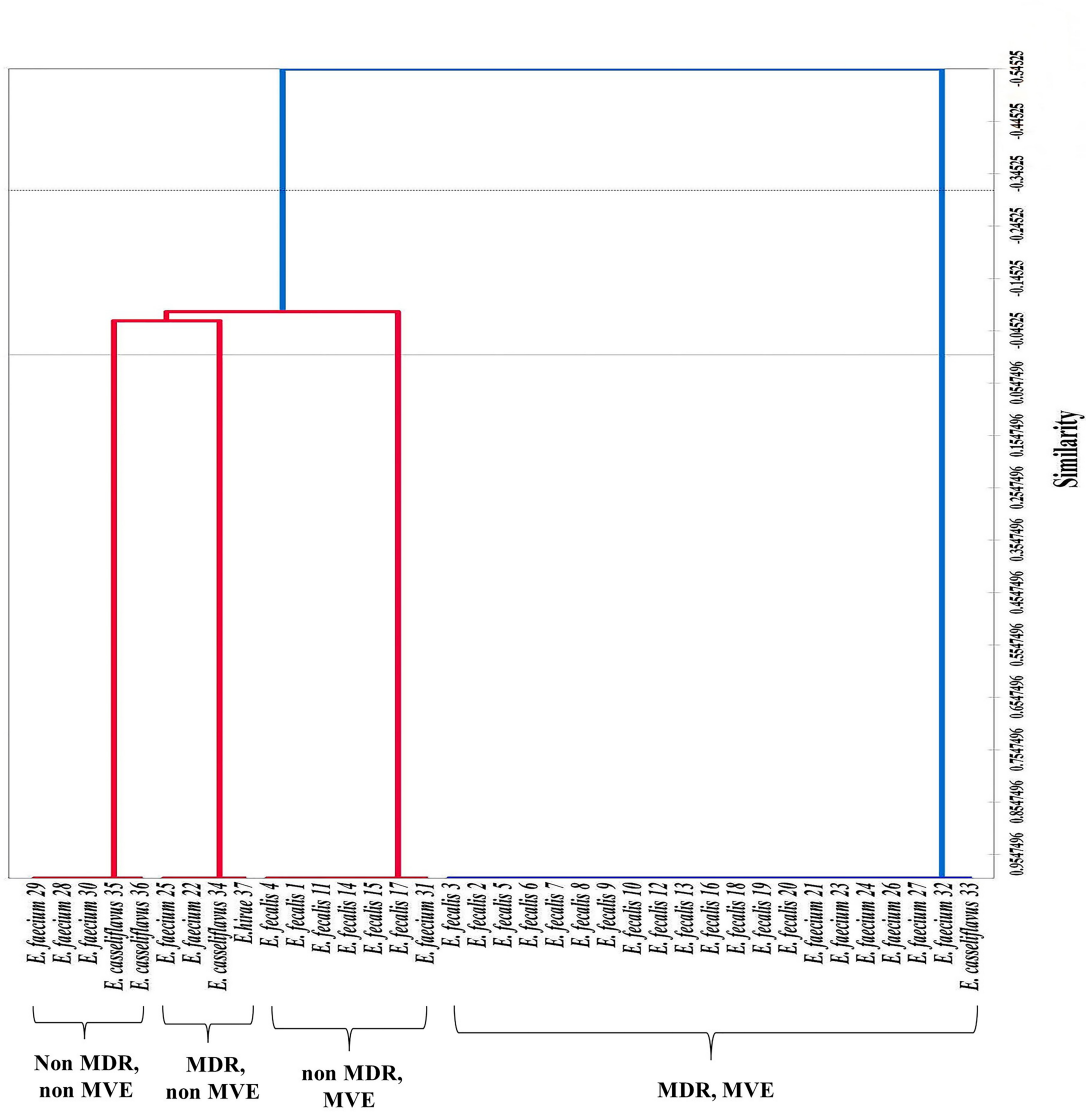
A dendrogram of 37 *Enterococcus* isolates originating from subclinical mastitic milk of sheep and goats was performed using the Pearson correlation coefficient based on their similarity analysis. Clustering was performed using the unweighted pair-group method.

Clustering the isolated *Enterococcus* strains according to the presence of MDR and/or virulence genes (Fig 5) revealed that 84% of MDR strains, which also represent almost half of the total number of isolates, were also MVE, and *E*. *faecalis* predominated this cluster.

## Discussion

Mastitis is the most persistent, prevailing, and costing infection affecting dairy animals worldwide [38]. In the present study, SCM was higher in goats than sheep. This finding was supported by Bourabah et al. [39], who stated that mastitis in goats is more often found in a subclinical form and is a critical pathological condition that causes a significant economic loss to goat farmers. Subclinical mastitis criteria such as somatic cell counts, CMT reactivity, and bacteriological culture outcomes are not as extensively described in small ruminants as in dairy cattle. Mastitis prevalence fluctuates from one study to another, and this might be due to the differences in areas, treatment procedures, management practices, and the microbial contamination of the surroundings.

Etiological agents should be identified to successfully manage and develop therapy and preventive techniques for mastitis [40]. The ratio concerning the existence of *Enterococcus* spp. is variable. Its ratio in mastitis cases ranged from 6–42% [5,29,30,41,42], which is lower than that obtained in this study (59%). However, Hamzah et al. [43] recorded a nearly comparable incidence of *Enterococcus* spp. in mastitic milk (60%). Consistently with what has been found by previous studies [29,30,42–45], *E*. *faecalis* was the dominant species, and the ratio of other species was variable. Nevertheless, *E*. *faecium* was the predominant isolated species by Kateete et al. [5], Gao et al. [6] and Cortes et al. [46]. The high ratio of *Enterococcus* spp. in this study may be attributed to the fact that *Enterococcus* spp. is considered an environmental pathogen that causes mastitis [4,27]. This high ratio might be due to management mistakes such as lack of farm cleanliness and sanitation, including poor ventilation and inadequate manure disposal [47].

*Enterococcus hirae* isolate was confirmed by 16Sr DNA sequencing after being failed to be identified by the VITEK system. Therefore, it is clear that in certain circumstances, biochemical identification systems such as the VITEK 2 fluorescent systems cannot be relied on solely. This situation highlighted the prominent irreplaceable role of 16S rDNA gene sequencing in the identification of species. The importance of the 16S rDNA sequencing is due to being not restricted to a specific group of bacteria (e.g., GenBank public databases). On the contrary, it covers the whole phylogenetic spectrum. Also, novel, not yet described species can be allocated to the most related bacterial group [48].

Treatment of SCM is deeply reliant on antibiotics. Cautious antibiotic therapy reduces the likelihood of progression into clinical form and prevents economic losses. Consuming food from various sources rich in bacterial populations will act as a donor or recipient of antibiotic resistance. The present concern is the acquired antibiotic resistance among *Enterococci*, which is highly difficult to medicate [49]. Therefore, it was critical to describe the antibiotic susceptibility/resistance profile among these isolates. The resistance of *Enterococcus* isolates obtained from subclinical mastitis varied among different studies [41,50,51].

Our findings confirmed the intrinsic resistance of *Enterococcus* spp. to β-lactams and acquired resistance to tetracyclines, erythromycin, linezolid, and vancomycin. The current results are mostly found higher than earlier published results [5,26,29,30,43,52–54]. However, for penicillin and oxacillin, our results were consistent with earlier studies [6,26,30]. Other studies reported higher results for tetracycline resistance [26,52,54]. Moreover, Ahmed et al. [30] and Citac et al. [52] reported higher incidences of resistance for erythromycin and linezolid.

The fact that *Enterococcus* spp. tend to overexpress Penicillin-binding proteins with low-affinity β-lactams, allowing them to be intrinsically resistant to penicillin or oxacillin [55,56], supports the strong resistance among the obtained isolates to β-lactams drugs in this study. *E*. *faecium* is inherently more resistant to antibiotics than *E*. *faecalis* [57], which is contrary to the displayed results of this study (Fig 2B). This result may be attributed to the ability *E*. *faecalis* to form thicker biofilms that create antibiotic tolerance [58,59], Similar results were reported by Cui et al. [60].

Since *Enterococcus* spp. may transmit antimicrobial-resistance or virulence genes easily via horizontal transmission, their existence in milk can promote the development of MDR strains, influencing the drug choice [6]. In this study, the association between *Enterococcus* spp. and resistance phenotypes varied. Unlike Cui et al. [60], a nearly identical incidence of MDR was detected among *E*. *faecalis* and *E*. *faecium* isolates. Nevertheless, we found *E*. *faecalis* strains disseminated across the six MDR patterns denoting that *E*. *faecalis* was more resistant than *E*. *faecium*. Also, β-lactams, vancomycin, and linezolid were the most prevalent antibiotics against which *Enterococcus* isolates were resistant and existed in all MDR patterns. This finding is consistent with Tatsing Foka and Ateba [61], who revealed that penicillin and vancomycin were the most frequent antimicrobials noticed in the different resistance patterns.

One of this study’s most concerning findings is the existence of high resistance to vancomycin and linezolid among *Enterococcus* isolates. Vancomycin-resistant *Enterococci* (VRE) is the leading cause of worldwide nosocomial infections, and its first emergence was recorded in 1908 in Great Britain. Since then, the VRE has been reported in different hospitals worldwide [62] and various environmental samples, including milk and dairy products [54]. VRE isolates represented 23% of intensive care unit (ICU) cases in Egypt, and 41.7% of them were suffering from renal disease, indicating a major issue in Egypt [63]. Also, the CDC reported 5,400 deaths out of 54,500 VRE infections among hospitalized patients in the United States in 2017 [64]. Furthermore, WHO listed VR *E*. *faecium* as a second category pathogen of high priority among antibiotic-resistant bacteria [65]. Therefore, the CDC stated VR *E*. *faecium* serious public health threat requiring immediate and continuous action in its report on the antibiotic resistance threats [64].

Regarding antimicrobial resistance for both vancomycin and linezolid together, this result agrees with Tatsing Foka and Ateba [61], who detected resistance to linezolid in 98% of VR *Enterococci*. Thus, the problem is more complicated now since linezolid is considered the last antibiotic resort for infections caused by MDR Gram-positive bacteria, including VRE [66].

The turnover of *Enterococci* spp. from harmless commensals to troublesome pathogens has risen over decades through various mechanisms such as the acquisition of resistant genes against several antibiotics from other organisms through transposons or chromosomal exchange, gene mutations, and modification of bacterial surface molecules [49] making the therapeutic options quite challenging. So, continuous genotypic screening of the resistant *Enterococcus* isolates is imperative for the food industry and public health [67]. PCR screening for antimicrobial resistance determinants in *Enterococcus* isolates originated from subclinical mastitic milk revealed that all the detected isolates carried one or more genes accountable for the antimicrobial resistance. Of all *Enterococcus* isolates studied, the *blaZ* and *ermB* genes were the most common antimicrobial resistance determinants. Woźniak-Biel et al. [7] reported lower results for *blaZ*, while Ahmed et al. [30] detected this gene in a higher frequency. According to Teuber et al. [68], the *ermB* gene is the most common macrolide resistance gene among *Enterococcus* spp. obtained from food. Several researchers detected higher percentages of *ermB* gene among their isolates from different regions [7,30,69,70], while Erbas et al. [43] and Cui et al. [60] found it at lower incidence. On the other hand, Gaglio et al. [22] could not detect the erythromycin resistance gene.

Vancomycin resistance encoded by the *vanA* gene could be found in a lower incidence of *Enterococcus* spp. isolated by [7,43,60,71]. In comparison, higher incidences of this gene were recorded [30], and none of *Enterococcus* spp. obtained by Gaglio et al. [22] and Cui et al. [60] harboured *vanA*. Regarding *tetM*, except for Tatsing Foka and Ateba [61] and Abdeltawab [71], who could not detect it in *Enterococcus* isolates, all the previously mentioned researchers reported a higher incidence of this gene. Additionally, a higher frequency of the *optrA* gene was reported by Ahmed et al. [30], and a lower one was reported by Cui et al. [60].

In Egypt, a number of antimicrobial agents such as β-lactams, aminoglycosides, glycopeptides, tetracyclines, phenicols, fluoroquinolones, lincosamides, polymyxins and sulfonamides have been used to control mastitis [47]. The misuse of these antimicrobials has been considered the main cause of antimicrobial resistance accumulation and contributes to introducing antimicrobial-resistant strains into the milk production system.

Biofilm has been implicated in the pathogenesis of enterococcal infection (e.g., mastitis) and contributed to recurrent infections [72–74]. Furthermore, biofilm is a significant source of contamination in the food processing sector as *Enterococcus* food isolates have the ability to produce biofilms [23,75]. Necidová et al. [76] recorded similar results to those obtained in this study which revealed that the biofilm, with its variable degree of intensity, was produced more frequently by *E*. *faecalis* isolates than other species. Furthermore, and consistent with this study, Elhadidy and Zahran [77] stated that there is a significant relationship between subclinical mastitis and biofilm formation; they reported that 18 of 38 biofilm-forming *E*. *faecalis* were isolated from clinical and subclinical mastitic milk. Also, they demonstrated a relationship between biofilm formation and its adherence to mammary gland epithelium, which aids in the recurrence of infection. In addition to the inherent resistance that *E*. *faecalis* exhibits against several antimicrobials, biofilm also enhances its resistance to these therapeutic agents. Therefore, it is considered a reservoir for resistance genes dissemination, making *E*. *faecalis* biofilm-related infections a clinical threat and extremely challenging to treat [27].

In the current study, the direct relation between the strength of biofilm formation and MDR addresses the genus *Enterococcus* as one of the most serious pathogens that could threaten health and life. Biofilm may lead to the acquisition of antimicrobial resistance by *Enterococci* through horizontal gene transfer. Acquired resistance in *Enterococci* generally occurs by exchanging pheromone-responsive genes, plasmids, or transposons [48]. The biofilm matrix enables communities of bacteria to be in close proximity [78]. It also provides an ideal reservoir for the cellular exchange of plasmids encoding for antibiotic resistance, thus potentially promoting bacterial resistance [79]. Horizontal transfer of resistance-conferring genes between bacterial cells within the biofilm and has been reported as being 700 times more efficient than among free-living, planktonic bacterial cells [80].

Subclinical mastitic milk containing MDR *Enterococci* is hazardous for consumers in case it is consumed raw because of its normal appearance. *Enterococci* with virulence determinants have the potential to cause a severe infection. As a result, another goal of the study was to evaluate the virulence degree of the isolated strains. Different incidence of genes that confer virulence to *Enterococcus* spp. have been recorded [18,19,22,53,59,60,81]. Genes encoding gelatinase (*gelE*) and collagen decomposition and adhesion (*ace*) were found in all *E*. *faecalis* isolates, forming the highest incidence among the total isolates. This result agrees with Ribeiro et al. [82] and Jiménez et al. [83], who stated that gelatinase is one of the most significant factors of virulence. Also, its encoding gene was frequently detected in isolates obtained from food such as raw, pasteurized milk and dairy products [20,53,84]. Gene *ace* was also found in *E*. *faecalis* isolates of food as well as a medical origin [20,85]. Similar to the presented results, Yoon and Lee. [53] detected *gelE* and *ace* in most *E*. *faecalis* isolates (99 and 94%, respectively), and *hyl* gene was not detected in any of the isolates either. The distribution of virulence genes among the isolated *Enterococcus* spp. revealed nine virulence profiles. These virulence profiles illustrated that *E*. *faecalis* predominated the most profiles. Also, it carried virulence genes more frequently than the other species, which is consistent with earlier results [15,19,53,59,86]. Our findings are supported by the fact that *E*. *faecalis* is the most frequent *Enterococcus* species associated with diseases [87].

In the present study, a highly significant correlation was observed between the ability of the obtained isolates to produce biofilm and the presence of *gelE*, and *ace* genes on the one hand, and between the same trait and the *EF3314* gene presence on the other hand. As seen in Fig 4, *gelE* and *ace* genes are associated with all biofilm phenotypes, while *EF3314* gene existence was related to almost 70% of all detected biofilm phenotypes. This result is consistent with previous results recorded by Mohamed et al. [88] that cleared the role of gelatinase in biofilm production. Other genetic manipulation studies verified that the *gelE* gene enhances biofilm formation [89–91]. Moreover, Cui et al. [60] found a strong correlation between biofilm production and the existence of the *ace* gene. Creti et al. [15] revealed that *EF3314* is a cell-anchor surface protein included in biofilm formation and involved in the early contact with epithelia.

Regarding the *esp* gene, the degree to which this gene contributes to biofilm formation has yielded inconclusive findings. Among the 27 biofilm-producing strains, only 10 strains possessed the *esp* gene, and it was absent in the rest of the biofilm-producing strains indicating the unsignificant relation between the presence of *esp* and biofilm formation. Elhadidy and Elsayyad [27] reported a similar observation. Contradictory findings concluded that the significant increase in biofilm formation might be attributed to *esp* gene expression [92,93]. Among the 17 *esp*-missing biofilm producers, 15 *gelE*-positive strains produced biofilm (including two strong and seven moderate), which agrees with Mohamed and Murray [94], who reported that *gelE* might contribute to biofilms formation in an *esp*-missing isolate.

The results of this study highlighted the hazards associated with *Enterococcus* spp. as an aetiology of SCM in sheep and goats. The isolated *Enterococcus* species can produce biofilm, which is a fundamental problem for dairy farms. Biofilm in the dairy farm affects the udder health and spreads to dairy processing plants through raw milk, establishing new biofilms designated as a risk for milk quality and consumer health [95]. Biofilm formation, in turn, is associated with acquiring multidrug resistance by a high incidence of *Enterococcus* spp. obtained in this study. The prevalence of virulence factors, along with high levels of resistance to a wide variety of antibiotics, reinforce the quite significant role of *Enterococci* spp., in particular *E*. *faecalis*, as opportunists in nosocomial infections. What makes this matter of more concern is that all MDR strains are resistant to both vancomycin and linezolid drugs that are crucial in the *Enterococcus* infections’ treatment. Another concern is using the subclinical mastitic milk containing MDR and virulent *Enterococcus* spp. in dairy products as they are a reservoir of virulence and antibiotic-resistant genes that could be transferred to humans microbiota in the food chain.

## Conclusion

The current study presents the ratio of *Enterococcus* spp. as a causative agent of subclinical mastitis in sheep and goats, their antimicrobial resistance and virulence profiles and their ability to produce biofilm. The results showed a high ratio of *Enterococcus* spp. in mastitic milk, highlighting the significance of milking hygiene practices. Also, a high ratio of antimicrobial resistance was found, especially for those considered important in human medicine like vancomycin and linezolid. 67.6% of the isolates were assigned as MDR. Likewise, a high frequency of biofilm formation and virulence genes were manifested among isolates. Moreover, biofilm production was correlated with the presence of virulence genes and the emergence of MDR strains. From this perspective, consumers must be aware of the health risks associated with raw milk or milk products consumption that may be a source of MDR and virulent enterococcal infections, especially *E*. *faecalis* and *E*. *faecium*. In this study, we suggest launching programs to teach the farmers the application of good hygienic practices and farm management and assure the need for more comprehensive governmental surveillance to monitor the use of antimicrobial agents by farmers. Finally, the last-resort antibiotics should not be used in veterinary medicine.

## Supporting information

S1 Table Primers sequences of antimicrobial resistance genes, amplicon sizes and cycling conditions. (DOCX)

S2 Table Primers sequences of virulence genes, amplicon sizes and cycling conditions. (DOCX)

S3 Table The accession number of registered *Enterococcus* isolates on Genbank. (DOCX)

## Abbreviations

SCM: Subclinical Mastitis
MDR: Multidrug resistance
VRE: Vancomycin-resistant *Enterococci*
MVE: Multi-virulent *Enterococci*
