# Supplementary material for: Antimicrobial resistance, virulence genes and biofilm formation in Enterococcus species isolated from milk of sheep and goat with subclinical mastitis
Source: PLoS One. 2021 Nov 15;16(11):e0259584. doi: 10.1371/journal.pone.0259584 (PMC8592430; doi:10.1371/journal.pone.0259584)
Supplement: S1 Table — (DOCX) [file pone.0259584.s001.docx]

**S1 Table. Primers sequences of antimicrobial resistance genes, amplicon sizes and cycling conditions.**

| Target gene | Primers sequences | Amplified segment (bp) | Primary  denaturation | Amplification (35 cycles) | | | Final extension | Reference |
| --- | --- | --- | --- | --- | --- | --- | --- | --- |
|  |  |  |  | Secondary denaturation | Annealing | Extension |  |  |
| *16S rDNA* | AGAGTTTGATCMTGGCTCAG | 1485 | 94˚C  5 min. | 94˚C  30 sec. | 56˚C  1 min. | 72˚C  1 min. | 72˚C  10 min. | [1] |
|  | TACGGYTACCTTGTTACGACTT |  |  |  |  |  |  |  |
| *blaZ* | ACTTCAACACCTGCTGCTTTC | 173 | 94˚C  5 min. | 94˚C  30 sec. | 54˚C  30 sec. | 72˚C  30 sec. | 72˚C  7 min. | [2] |
|  | TGACCACTTTTATCAGCAACC |  |  |  |  |  |  |  |
| *ermB* | CATTTAACGACGAAACTGGC | 425 | 94˚C  5 min. | 94˚C  30 sec. | 51˚C  40 sec. | 72˚C  45 sec. | 72˚C  10 min. | [3] |
|  | GGAACATCTGTGGTATGGCG |  |  |  |  |  |  |  |
| *vanA* | CATGACGTATCGGTAAAATC | 885 | 94˚C  5 min. | 94˚C  30 sec. | 50˚C  40 sec. | 72˚C  50 sec. | 72˚C  10 min. | [4] |
|  | ACCGGGCAGRGTATTGAC |  |  |  |  |  |  |  |
| *tetM* | GTG GAC AAA GGT ACA ACG AG | 406 | 94˚C  5 min. | 94°C  1 min. | 55°C  1 min. | 72°C  1 min. | 72˚C  10 min. | [5] |
|  | CGG TAA AGT TCG TCA CAC AC |  |  |  |  |  |  |  |
| *optrA* | AGGTGGTCAGCGAACTAA | 1395 | 94˚C  5 min. | 94˚C  30 sec. | 53˚C  1 min. | 72˚C  1 min. | 72˚C  12 min. | [6] |
|  | ATCAACTGTTCCCATTCA |  |  |  |  |  |  |  |

Primers used were supplied from Metabion (Germany)

References

1. Lagacé L, Pitre M, Jacques M, Roy D. Identification of the bacterial community of Maple Sap by using amplified ribosomal DNA (rDNA) restriction analysis and rDNA sequencing. Appl Environ Microbiol. 2004;70: 2052–2060. doi:10.1128/aem.70.4.2052-2060.2004
2. Duran N, Ozer B, Duran GG. Onlen Y, Demir C. Antibiotic resistance genes & susceptibility patterns in *Staphylococci*. Indian J Med Res. 2012; 135, pp 389-396.
3. Schlegelova J, Vlkova H, Babak V, Holasova M, Jaglic Z, Stosova T, et al. Resistance to erythromycin of *Staphylococcus* spp. isolates from the food chain. Vet Med (Praha). 2008;53: 307–314. doi:10.17221/1856-vetmed
4. Patel R, Uhl JR, Kohner P, Hopkins MK, Cockerill FR. Multiplex PCR detection of *vanA*, *vanB*, *vanC-1*, and *vanC-2/3* genes in *Enterococci*. J Clin Microbiol. 1997;35: 703–707. doi:10.1128/jcm.35.3.703-707.1997
5. Ng L-K, Martin I, Alfa M, Mulvey M. Multiplex PCR for the detection of tetracycline resistant genes. Mol Cell Probes. 2001;15: 209–215. doi:10.1006/mcpr.2001.0363
6. Wang Y, Lv Y, Cai J, Schwarz S, Cui L, Hu Z, et al. A novel gene, *optrA*, that confers transferable resistance to oxazolidinones and phenicols and its presence in *Enterococcus* *faecalis* and *Enterococcus* *faecium* of human and animal origin. J Antimicrob Chemother. 2015;70: 2182–2190. doi:10.1093/jac/dkv116
