# Supplementary material for: Antimicrobial resistance, virulence genes and biofilm formation in Enterococcus species isolated from milk of sheep and goat with subclinical mastitis
Source: PLoS One. 2021 Nov 15;16(11):e0259584. doi: 10.1371/journal.pone.0259584 (PMC8592430; doi:10.1371/journal.pone.0259584)
Supplement: S2 Table — (DOCX) [file pone.0259584.s002.docx]

**S2 Table. Primers sequences of virulence genes, amplicon sizes and cycling conditions.**

| Target gene | Primers sequences | Amplified segment (bp) | Primary  denaturation | Amplification (35 cycles) | | | Final extension | Reference |
| --- | --- | --- | --- | --- | --- | --- | --- | --- |
|  |  |  |  | Secondary denaturation | Annealing | Extension |  |  |
| *EF3314* | AGAGGGACGATCAGATGAAAAA | 566 | 94˚C  5 min. | 94˚C  30 sec. | 55˚C  40 sec. | 72˚C  45 sec. | 72˚C  10 min. | [1] |
|  | ATTCCAATTGACGATTCACTTC |  |  |  |  |  |  |  |
| *ace* | GGAATGACCGAGAACGATGGC | 616 | 94˚C  5 min. | 94˚C  30 sec. | 58˚C  40 sec. | 72˚C  45 sec. | 72˚C  10 min. |  |
|  | GCTTGATGTTGGCCTGCTTCCG |  |  |  |  |  |  |  |
| *cylA* | ACTCGGGGATTGATAGGC | 688 | 94˚C  5 min. | 94˚C  30 sec. | 50˚C  40 sec. | 72˚C  45 sec. | 72˚C  10 min. | [2] |
|  | GCTGCTAAAGCTGCGCTT |  |  |  |  |  |  |  |
| *gelE* | TATGACAATGCTTTTTGGGAT | 213 | 94˚C  5 min. | 94˚C  30 sec. | 50˚C  30 sec. | 72˚C  30 sec. | 72˚C  7 min. |  |
|  | AGATGCACCCGAAATAATATA |  |  |  |  |  |  |  |
| *hyl* | ACAGAAGAGCTGCAGGAAATG | 276 | 94˚C  5 min. | 94˚C  30 sec. | 55˚C  30 sec. | 72˚C  30 sec. | 72˚C  7 min. |  |
|  | GACTGACGTCCAAGTTTCCAA |  |  |  |  |  |  |  |
| *Esp* | AGATTTCATCTTTGATTCTTGG | 510 | 94˚C  5 min. | 94˚C  30 sec. | 50˚C  40 sec. | 72˚C  45 sec. | 72˚C  10 min. |  |
|  | AATTGATTCTTTAGCATCTGG |  |  |  |  |  |  |  |
| *Asa1* | GCACGCTATTACGAACTATGA | 375 | 94˚C  5 min. | 94˚C  30 sec. | 50˚C  40 sec. | 72˚C  45 sec. | 72˚C  10 min. |  |
|  | TAAGAAAGAACATCACCACGA |  |  |  |  |  |  |  |

Primers used were supplied from Metabion (Germany)

References

1. Creti R, Imperi M, Bertuccini L, Fabretti F, Orefici G, Di Rosa R, et al. Survey for virulence determinants among Enterococcus faecalis isolated from different sources. J Med Microbiol. 2004;53: 13–20. doi:10.1099/jmm.0.05353-0
2. Vankerckhoven V, Van Autgaerden T, Vael C, Lammens C, Chapelle S, Rossi R, et al. Development of a multiplex PCR for the detection of *asa1*, *gelE*, *cylA*, *esp*, and *hyl* genes in *Enterococci* and survey for virulence determinants among European hospital isolates of *Enterococcus* *faecium*. J Clin Microbiol. 2004;42: 4473–4479. doi:10.1128/JCM.42.10.4473-4479.2004
