# Supplementary material for: Antimicrobial resistance, virulence genes and biofilm formation in Enterococcus species isolated from milk of sheep and goat with subclinical mastitis
Source: PLoS One. 2021 Nov 15;16(11):e0259584. doi: 10.1371/journal.pone.0259584 (PMC8592430; doi:10.1371/journal.pone.0259584)
Supplement: S3 Table — (DOCX) [file pone.0259584.s003.docx]

**S3 Table. Accession number of the registered *Enterococcus* isolates on Genbank.**

| Species | Accession number |
| --- | --- |
| *E. faecalis* | MW642507 |
| *E. faecium* | MW644528 |
| *E, casseliflavus* | MW644643 |
| *E. hirae* | MW644529 |
